# Pancreatic Steatosis as a Risk Factor for Pancreatic Ductal Adenocarcinoma: Pathogenesis and Clinical Implications

**DOI:** 10.14309/ctg.0000000000000832

**Published:** 2025-02-24

**Authors:** Zoi Papalamprakopoulou, Prasenjit Dey, Rachel Frascati, Christos Fountzilas

**Affiliations:** 1 Division of Gastroenterology, Hepatology, and Nutrition, Department of Medicine, Jacobs School of Medicine and Biomedical Sciences, University at Buffalo, Buffalo, New York, USA;; 2 Division of Gastrointestinal Medical Oncology, Department of Medicine, Roswell Park Comprehensive Cancer Center, Buffalo, New York, USA;; 3 Department of Immunology, Roswell Park Comprehensive Cancer Center, Buffalo, New York, USA;; 4 Strategy, Business Development & Outreach, Roswell Park Comprehensive Cancer Center, Buffalo, New York, USA.

**Keywords:** pancreatic steatosis, fatty pancreas, pancreatic ductal adenocarcinoma, pancreatic cancer, surveillance, risk factors

## Abstract

Pancreatic steatosis is defined as the ectopic accumulation of fat in the pancreas. While historically considered a benign incidental imaging finding, it is now recognized as a significant and potentially reversible risk factor for pancreatic ductal adenocarcinoma (PDAC) independent of obesity. Although its epidemiology is not well characterized, meta-analysis data suggest an approximately 30% prevalence, with individual studies reporting even higher rates among patients with obesity and/or metabolic syndrome. Concurrently, PDAC incidence is rising and is projected to soon become the second leading cause of cancer-related deaths. Given the critical importance of early PDAC detection and intervention for improving survival, it is particularly timely to explore the associations between pancreatic steatosis and PDAC. This review aims to provide a comprehensive overview of the pathogenesis and clinical associations between pancreatic steatosis and PDAC and to discuss future perspectives within the context of current PDAC surveillance practices.

## Graphical abstract

**Figure:**
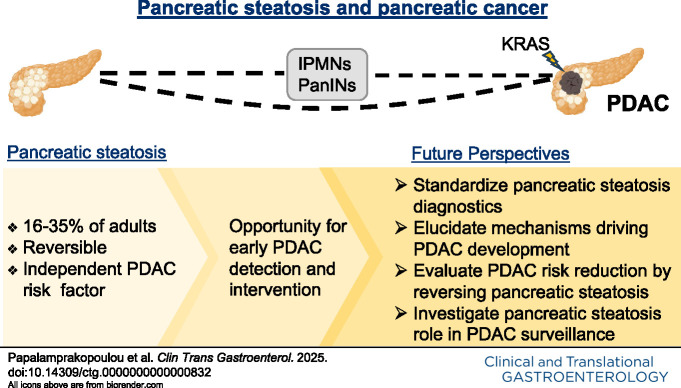

## INTRODUCTION

Pancreatic ductal adenocarcinoma (PDAC) accounts for over 90% of all pancreatic malignancies (1). It is projected to become the second most common cause of cancer-related death by 2030 (2). The 5-year overall survival (OS) rate for PDAC is 13.0%, varying significantly between stages (3). Unfortunately, most PDAC cases are unresectable or metastatic on diagnosis, where the median OS is approximately 11 months with chemotherapy (4). By contrast, surgical resection with adjuvant chemotherapy for stage I PDAC has a 5-year OS of 38.2%, underscoring the critical importance of early diagnosis and intervention (5).

The etiology of PDAC remains largely unknown (6). Increased KRAS oncogene activity is the primary regulator of PDAC initiation and maintenance, implicated in over 90% of cases, whereas additional mutations, such as the inactivation of TP53, CDKN2A/p16, and SMAD4 tumor suppressor genes, drive disease progression (7,8). Only 5%–10% of PDAC cases are related to inherited genetic factors (9). Familial pancreatic cancer, defined by having at least 2 first-degree relatives with the disease without known susceptibility genes, increases the risk by 6.79 fold (10). Hereditary syndromes include hereditary breast and ovarian cancer syndrome, Lynch syndrome, and Peutz-Jeghers syndrome (11). Nonhereditary nonmodifiable risk factors include age, male sex, race (higher in African Americans), and non-O blood group (12–15). Modifiable risks include smoking (increases risk by 75%), obesity (increases risk by 60%), diabetes, metabolic syndrome, high alcohol consumption, pancreatitis, and metabolic dysfunction-associated steatotic liver disease (MASLD), previously known as nonalcoholic fatty liver disease (NAFLD) (2,16–27).

Pancreatic steatosis has been recently identified as an independent risk factor for PDAC (28–30). Historically, pancreatic steatosis, or ectopic fat accumulation in the pancreas, was considered a benign incidental imaging finding that did not require further investigation. Epidemiologic data remain limited, with prevalence varying by diagnostic methods and ethnicity. Studies report a 16% prevalence in Taiwan, 35% in Jakarta, and 27.8% in the United States (31–33). A meta-analysis of 11 studies, totaling 12,675 individuals, with the majority conducted among Asian populations, showed a 33% prevalence (34). Pancreatic steatosis also affects 10% of children and is related to obesity (35).

The high prevalence of pancreatic steatosis and its recognition as a risk factor for PDAC necessitate further investigation into its pathogenesis, connections to cancer development, and its role in cancer surveillance. Understanding these factors may present opportunities for early PDAC detection and intervention. This review aims to provide a comprehensive overview of pancreatic steatosis, focusing on its associations with pancreatic carcinogenesis, with the goal of identifying opportunities for early PDAC diagnosis.

## PANCREATIC STEATOSIS

### Nomenclature

Pancreatic steatosis refers to the accumulation of fat in the pancreas. Several synonyms for pancreatic steatosis are reported in the literature, including fatty pancreas, pancreatic lipomatosis, and pancreatic lipidosis. Lipomatous pseudohypertrophy represents an extreme variant of pancreatic fat accumulation, characterized by pancreatic enlargement and replacement of the exocrine system by fat, with no association with obesity (36). Pancreatic steatosis mainly involves the interlobular and intralobular spaces, usually sparing the intra-acinar space, where the exocrine cells reside and the endocrine cells (37,38). By contrast, in MASLD, lipid accumulation predominately occurs intracellularly within hepatocytes (39). Nonalcoholic fatty pancreas disease refers to fat accumulation related to obesity and/or metabolic syndrome in the absence of significant alcohol intake. Whether the nomenclature for nonalcoholic fatty pancreas disease should be updated to metabolic dysfunction-associated steatotic pancreas disease, in line with the recent shift from NAFLD to MASLD, remains an open question (40).

### Associations with hepatic steatosis

There is a significant and mechanistically complex overlap between pancreatic and hepatic steatosis through a bidirectional relationship thought to be mediated by obesity (41–43). Pancreatic steatosis is significantly associated with MASLD and biopsy-proven metabolic dysfunction-associated steatohepatitis, with the pancreas being more susceptible to fat deposition than the liver, often preceding hepatic steatosis (31,44). Lee et al (45) reported a 68% prevalence of hepatic steatosis among patients with pancreatic steatosis, whereas the prevalence of pancreatic steatosis among patients with hepatic steatosis was 97%. However, a recent study found no correlation between pancreatic steatosis and MASLD (46).

### Pathogenesis and risk factors

Pancreatic steatosis is thought to arise through either “fatty infiltration” or “fatty replacement” pathways (47).

#### Fatty infiltration pathway.

“Fatty infiltration” occurs when adipocytes infiltrate the pancreatic parenchyma in association with obesity and/or metabolic syndrome, a reversible process (34,42,48). Obesity-induced inflammation, reduced anti-inflammatory cytokines such as interleukin (IL)-10 (38,49), pancreatic islet inflammation (50), endoplasmic reticulum (ER) stress (51), and circadian changes are believed to contribute to its pathogenesis (52). Sex and hormonal changes also influence pancreatic fat content, which increases throughout childhood and plateaus around 50 years old (53). Before age 50 years, obese men tend to have higher pancreatic fat content compared with obese women, but this prevalence equalizes after menopause and becomes similar to men after age 55 years (54–57).

Sepe et al showed a 37% higher prevalence of fatty infiltration among individuals with metabolic syndrome components, such as body mass index ≥30, hyperlipidemia, type 2 diabetes mellitus, and hypertension, with a similar trend observed in pediatric populations, where prevalence is more than twice as common in obese (19.0%) compared with nonobese (8.0%) children (33,35). Additional studies link the severity of pancreatic fatty infiltration to older age, high waist circumference, high serum triglycerides, high low-density lipoprotein cholesterol, low serum amylase, hemoglobin A1c levels >6, and MASLD (57–59).

#### Fatty replacement pathway.

“Fatty replacement” occurs when acinar cell die because of noxious stimuli and are replaced by adipocytes, an irreversible process (47). Possible mechanisms include drugs (corticosteroids, gemcitabine, rosiglitazone, octreotide), toxins, alcohol, infections (HIV, hepatitis B virus, *Helicobacter pylori*, reovirus), congenital disorders (cystic fibrosis, hereditary pancreatitis, Shwachman-Diamond syndrome, Johanson-Blizzard syndrome), chronic pancreatitis, iron overload, and malnutrition (33,55–57,60,61).

### Diagnosis

There is no well-recognized threshold to determine the upper limit of normal pancreatic fat, and there is a lack of standardized criteria for diagnosing pancreatic steatosis and grading its severity. Although there is no gold-standard imaging modality for diagnosis, magnetic resonance imaging (MRI) is generally considered the most effective. Wong et al suggested a 10.4% upper limit of normal pancreatic fat on MRI, whereas Singh et al proposed a cutoff of 6.2% (34,55). In adolescents, a cutoff of 5.0% has been suggested (62). However, computed tomography (CT) is more commonly performed as part of pancreatic cancer evaluation, with many studies using a pancreas-to-spleen attenuation ratio of less than 0.7 as a predictor of pancreatic steatosis presence (63–65). For ultrasound, the following grading system has been suggested: echogenicity of the pancreas slightly higher than the kidney is grade I, echogenicity of the pancreas significantly higher than the kidney but lower than the retroperitoneal fat is grade II, and echogenicity of the pancreas equal to or higher than the retroperitoneal fat is grade III (60).

## CLINICAL EVIDENCE LINKING PANCREATIC STEATOSIS TO PDAC

Table 1 summarizes key clinical studies that evaluated the association of pancreatic steatosis with PDAC (Table 1).

**Table 1.** Key clinical studies that evaluated the association of pancreatic steatosis with PDAC

Study	Study design	Participant number (N)	Country	Inclusion criteria	Exclusion criteria	Diagnostic modality for pancreatic steatosis	Key findings
Hori et al (2014) (66)	Case-control	187	Japan	Cases: PDAC (N = 102) Controls: without PDAC (N = 85)	Pancreatitis Sections with >30% fibrous area	Histopathology	Pancreatic steatosis was significantly associated with PDAC (OR 6.1, *P* < 0.001) Higher pancreatic steatosis in PDAC compared with noncancerous controls (26% vs 15%, *P* < 0.001)
Tomita et al (2014) (67)	Case-control	174	Japan	Cases: PDAC (N = 76) Controls: without PDAC (N = 98)	Pancreatitis	Histopathology Pancreatic steatosis was categorized as follows: • Grade 0 (0%–1% steatosis) • Grade 1 (2%–5%) • Grade 2 (6%–10%) • Grade 3 (11%–15%) and grade 4 (>16%)	PDAC cases showed significantly higher rates than controls for: Pancreatic steatosis >5% (72% vs 44%, *P* = 0.0002) Fibrosis >10% (86% vs 42%, *P* < 0.0001) Inflammatory cell infiltration >5% (14% vs 3%, *P* = 0.0061)
Mathur et al (2009) (38)	Case-control	40	United States	Patients who underwent resection for PDAC Cases: lymph node–positive (N = 20) Controls: lymph node–negative (N = 20)	N/A	Histopathology	Lymph node–positive PDAC cases had significantly more pancreatic steatosis compared with controls (46.4 ± 8.7 vs 21.4 ± 4.8; *P* < 0.02) and decreased fibrosis (1.7 ± 0.3 vs 2.7 ± 0.3; *P* < 0.02) Reduced mean survival among (18.9 ± 2.7 vs 30.8 ± 4.8 mo, *P* = 0.04)
Mathur et al (2011) (69)	Retrospective cohort	42	Unites States	Patients who underwent pancreatectomy for PDAC	N/A	CT Pancreatic steatosis was defined as pancreatic attenuation <30 HU	Significantly higher percentage of patients with pancreatic steatosis had positive lymph nodes (70% vs 42%, *P* < 0.002) No survival differences between patients with and without pancreatic steatosis (18 ± 11 vs 16 ± 15 mo, *P* = 0.7)
Lesmana et al (2018) (32)	Retrospective cohort	162	Indonesia	Patients who underwent EUS (26.5% with pancreatic cancer, 32.7% with pancreatic steatosis)	Other pancreatic disease Significant alcohol consumption Primary cancer with pancreatic metastasis	EUS Pancreatic steatosis was defined as hyperechoic pancreas compared with kidney	Pancreatic steatosis was significantly associated pancreatic cancer (OR 18.027, *P* = 0.001)
Khoury and Sbeit (2022) (72)	Retrospective cohort	519	Israel	Patients older than 18 yr old who underwent EUS for hepatobiliary indications (48 with pancreatic cancer)	N/A	EUS Pancreatic steatosis was defined as hyperechoic pancreas compared with kidney	Pancreatic steatosis was significantly associated with pancreatic cancer (OR 2.35, 95%, *P* = 0.04)
Sbeit et al (2021) (73)	Retrospective cohort	569	Israel	Patients older than 18 yr who underwent EUS for hepatobiliary indications (50 with PDAC, 13.7% with pancreatic steatosis)	Significant alcohol consumption	EUS	Pancreatic steatosis was not significantly associated with PDAC (OR 1.07, *P* = 0.15)
Mandai et al (2019) (74)	Retrospective cohort	115	Japan	Patients who underwent EUS for IPMN at the initial detection and with a medical history of high risk for pancreatic cancer (23 with IPMN and PDAC, 92 with IPMN without PDAC)	Patients who underwent EUS or US for the first time to diagnose PDAC	EUS Pancreatic steatosis was defined as hyperechoic pancreas compared with left kidney or spleen	Hyperechogenic pancreas was associated with PDAC concomitant with IPMN (OR 7.07, *P* = 0.01)
Fukuda et al (2017) (65)	Retrospective cohort	183	Japan	Patients who underwent distal pancreatectomy (75 with PDAC, 14 with pancreatic steatosis)	Insufficient CT assessment	Histopathology CT (evaluated PI, calculated as P/S ratio) Pancreatic steatosis was defined as a PI ≤ 0.70	PI was significantly lower in the pancreatic steatosis group (0.51 vs 0.83, *P* = 0.0049) PI ≤ 0.70 was significantly associated with PDAC (OR 2.31, *P* = 0.023) No significant associations between the PI and fibrosis (*P* = 0.15) or inflammatory cell infiltration (*P* = 0.90)
Fukui et al (2019) (79)	Retrospective cohort	55	Japan	Patients who underwent pancreatectomy, had preoperatively undergo MRI-PDFF using IDEAL-IQ and unenhanced CT, and those who had not receive preoperative therapy (24 with pancreatic cancer, 31 with nonpancreatic cancer tumors)	Pancreatic atrophy with extensive pancreatic ductal dilation Lack of histologically analyzable nontumorous pancreatic parenchyma	Histopathology MRI (PDFF) CT (PI) Pancreatic steatosis was defined as a MRI-PDFF >7.10% Pancreatic steatosis was defined as a PI <0.66	Pancreatic cancer group had higher MRI-PDFF and histologic pancreatic fat fraction (*P* < 0.01) but lower PI (*P* < 0.01) MRI-PDFF was the only independent risk factor for pancreatic cancer (OR 1.19, *P* < 0.01)
Yamazaki et al (2024) (28)	A. Observational prospective cohort B. Mendelian randomization study	A. 29,463 B. 25,617	United Kingdom	A. UK Biobank participants who underwent pancreas MRI (11,485 had a high IPFD of >10%, and 17,978 had a low IPFD ≤10%). Median follow-up = 4.5 yr B. Eight of nine IPFD-associated genetic variants from a genome-wide association study in the UK Biobank	N/A	MRI High-IPFD was defined as mean IPFD value >10% Low-IPFD was defined as mean IPFD value ≤ 10%	A. IPFD >10% was significantly associated with PDAC (HR 3.35, *P* = 0.001). PDAC cumulative incidence was 0.28% in the high IPFD and 0.07% in the low-IPFD group B. Genetically determined IPFD is associated with PDAC (OR, 2.46, *P* = 0.002) in the Pancreatic Cancer Cohort Consortium I, II, III/Pancreatic Cancer Case-Control Consortium data set (8,275 PDAC cases and 6,723 noncases)
Desai et al (2020) (78)	Case-control	303	United States	Cases: 68 PDAC with available noncontrast CT before diagnosis Controls: 235 without history of malignancy in last 10 yr, who underwent CT colonography for cancer screening	Known genetic syndrome with an increased pancreatic cancer risk Pancreatobiliary pathology	Noncontrast CT (evaluated P.S100; measure of the difference between pancreatic and splenic attenuation) A lower P.S100 indicated higher pancreatic steatosis	Pancreatic steatosis was higher in PDAC cases (lower P.S100 in cases [86.452] vs controls [92.414]; *P* = 4.016e-06) Pancreatic steatosis was significantly associated with PDAC (OR 3.75, *P* = 0.000171)
Hoogenboom et al (2021) (64)	Case-control	149	United States	Cases: 32 patients with PDAC who underwent noncontrast CT 1 mo to 3 yr before PDAC diagnosis Controls: 117 patients with noncontrast CT who did not develop PDAC within 3 yr after imaging	Cases: prediagnostic CT showed an obvious pancreatic mass, CT was conducted within 4 wk after abdominal surgery, and PDAC was a recurrence or arose from a mucinous cystic neoplasm, history of pancreatic surgery, or splenectomy Controls: lost to follow-up within 3 yr after imaging, CT was conducted within 4 wk after abdominal surgery, as well as in individuals with a history of pancreatic or extrahepatic biliary malignancy and history of pancreatic surgery or splenectomy	Noncontrast CT (evaluated P/S ratio and P − S difference) Pancreatic steatosis was defined as a P/S ratio <0.70	Pancreatic steatosis on prediagnostic CT was found in 71.9% of PDAC cases and in 45.3% of controls (OR 3.09, *P* = 0.0094) Pancreatic steatosis was independently associated with PDAC (OR 2.7, *P* = 0.037) P/S and P − S differences more pronounced ≤6 mo before the PDAC diagnosis

CT, computed tomography; EUS, endoscopic ultrasound; HR, hazard ratio; HU, Hounsfield units; IPFD, intrapancreatic fat deposition; IPMN, intraductal papillary mucinous neoplasm; MRI-PDFF, magnetic resonance imaging proton density fat fraction; OR, odds ratio; P − S, difference between pancreatic and splenic attenuation; P/S, pancreas-to-spleen attenuation; PDAC, pancreatic ductal adenocarcinoma; PI, pancreatic index; US, ultrasound.

### Pancreatectomy specimens

Research studies evaluating pancreatectomy specimens histologically have consistently demonstrated that patients with PDAC exhibit significantly higher levels of pancreatic steatosis compared with controls (66,67). Pancreatic steatosis has been established as an independent risk factor for PDAC, beyond the effects of obesity, with risk increasing with steatosis severity (66). In addition to steatosis, fibrosis and inflammatory cell infiltration are prominent histological features in PDAC cases (67). A study revealed that lymph node–positive PDAC cases had higher levels of pancreatic steatosis and less fibrosis compared with lymph node–negative controls postpancreatoduodenectomy, and findings also observed in preoperative CT scans (68,69). These differences were associated with poorer survival, supporting the hypothesis that pancreatic fat influences the tumor microenvironment to promote tumor spread (70).

Furthermore, the histological evaluation of pancreatic tissue reveals a link between pancreatic steatosis and precancerous lesions. Pancreatic intraepithelial neoplasia (PanIN) has been significantly associated with intralobular fat and fibrosis, indicating the role of steatosis in early carcinogenesis (71). Obesity has also been correlated with a higher prevalence of PanIN lesions. These findings highlight the complex interplay between pancreatic steatosis, obesity, early carcinogenesis, and the progression of precancerous lesions to malignancy.

### Imaging studies

In accordance with histological evaluations of pancreatectomy specimens, imaging studies also identify pancreatic steatosis as a significant risk factor for PDAC. Retrospective analyses of endoscopic ultrasound (EUS) patient records consistently highlight pancreatic steatosis as the sole significant risk factor for PDAC (32,72). Significant associations have also been observed in EUS data between pancreatic steatosis and MASLD, gallstones, intraductal papillary mucinous neoplasms (IPMNs), and IPMNs coexisting with PDAC (73,74).

CT and MRI findings further suggest that pancreatic steatosis could serve as an early biomarker for malignancies developing within IPMNs (75–77). On CT scans, lower pancreatic density, referred to as the pancreatic index (PI), has been shown to predict pancreatic steatosis with a 79% sensitivity and specificity. Both a lower PI and P.S100, another CT proxy for pancreatic steatosis, have been shown to serve as independent predictors of PDAC (65,78). While some researchers suggest that pancreatic fat measured on MRI may be a superior imaging biomarker for pancreatic cancer compared with the PI of CT, they also acknowledge the limitations of MRI in routine clinical practice (79).

Studies evaluating abdominal CT scans before PDAC diagnosis suggest that pancreatic steatosis may precede the development of PDAC, observing stable pancreatic steatosis for up to 3 years before cancer diagnosis that indicates a potential temporal relationship (64,78). A prospective study using MRI data from 29,463 participants demonstrated that severe pancreatic steatosis (>10%) tripled the risk of PDAC over a 4.5-year follow-up, further supporting a causal relationship between pancreatic steatosis and PDAC (28). In addition, meta-analysis data from 13 studies, encompassing 2,178 patients, revealed a pooled prevalence of pancreatic steatosis at 52.0% among individuals with PDAC or premalignant pancreatic lesions (59). Another meta-analysis of 16 primarily Japanese retrospective studies, involving 2,956 participants, found that pancreatic steatosis is approximately 6 times more prevalent in patients with PDAC compared with those without PDAC (29).

## MECHANISMS LINKING PANCREATIC STEATOSIS TO PDAC

**Figure 1.**
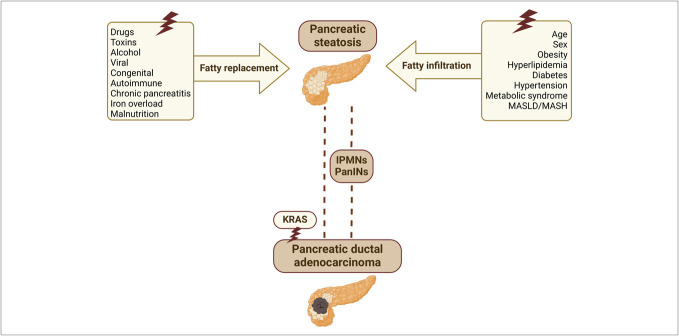
Pathogenesis of pancreatic steatosis and its associations with PDAC. Pancreatic steatosis is believed to develop through 2 primary mechanisms: fatty replacement and the fatty infiltration pathway. Research suggests that pancreatic steatosis is associated with an increased risk of PDAC, both directly and indirectly, through the development of high-risk lesions, such as intraductal papillary mucinous neoplasms and pancreatic intraepithelial neoplasia. Pancreatic steatosis-induced oncogenic KRAS signaling, implicated in nearly all PDAC cases, may contribute to steatosis-associated PDAC development. IPMN, intraductal papillary mucinous neoplasm; MASLD/MASH, metabolic dysfunction-associated steatotic liver disease/metabolic dysfunction-associated steatohepatitis; PanINs, pancreatic intraepithelial neoplasias; PDAC, pancreatic ductal adenocarcinoma.

Despite evidence linking pancreatic steatosis to precancerous pancreatic lesions and PDAC (Figure 1), the mechanisms by which it contributes to pancreatic carcinogenesis remain unclear. The KRAS mutation, a genetic event present in most of the PDAC cases, requires a second hit, such as the loss of tumor suppressor genes, for cancer initiation and progression (80). The following nongenetic factors related to pancreatic steatosis could serve as activating events for oncogenic KRAS signaling, contributing to pancreatic steatosis-induced PDAC, with current hypotheses focusing on the roles of inflammatory pathways and the tumor microenvironment (81).

### Adipokines and inflammatory pathways

In pancreatic steatosis, proinflammatory adipokines are released systemically from white adipose tissue and locally in the pancreas (82,83). Both obesity and PDAC are linked to increased leptin levels and decreased adiponectin levels (84). A study on Syrian golden hamsters showed that *N*-nitroso*bis*(2-oxopropyl)amine treatment induces severe pancreatic steatosis and robust production of cytokines and adipokines and accelerates PDAC development (85). Leptin promotes a low-grade inflammatory state by upregulating IL-1, IL-6, tumor necrosis factor-a, leukotriene B4, and cyclooxygenase-2, promoting a T-helper 1 phenotype, suppressing regulatory T cells, and upregulating Notch signaling, which further promotes PDAC development (86,87). Adiponectin antagonizes leptin signaling and inhibits carcinogenesis through mitogen-activated protein kinase, mechanistic target of rapamycin, phosphoinositide 3-kinase/protein kinase B, nuclear factor kappa B, and sphingolipid metabolic pathways (88,89). In addition, leptin activates the JAK2/STAT3 signaling cascade in PDAC cells, which is suppressed by adiponectin. These interactions, which promote low-grade inflammation driven by adipokines in pancreatic steatosis, may represent a critical mechanism underlying PDAC initiation and progression.

### Renin-angiotensin system

Adipocytes produce angiotensin-II (A-II), which regulates lipid synthesis and storage (90,91). In obesity, increased A-II and angiotensinogen contribute to adipose inflammation, glucose intolerance, and insulin resistance (92,93). Experimental studies show that overactivation of the renin-angiotensin system induces ER stress, promoting a pro-inflammatory phenotype in pancreatic cells which may contribute to pancreatic cancer initiation (93,94). A-II also regulates pancreatic tumor growth, angiogenesis, and metastasis (95). A study of 8,158 patients with PDAC showed improved survival with A-II receptor blockers, and a clinical trial found a 61% R0 resection rate with losartan and folinic acid, fluorouracil, irinotecan, and oxaliplatin chemotherapy (96–98). These findings suggest that A-II may increase the risk of PDAC risk by driving inflammation while promoting tumor growth and metastasis, highlighting its potential as a promising therapeutic target.

### Pancreatic stellate cells

Pancreatic stellate cells (PaSCs) are myofibroblast-like cells residing in a quiescent state in the exocrine pancreas, involved in tissue repair and fibrosis in chronic pancreatitis, PanIN lesions, and PDAC (99,100). When activated by inflammatory cytokines, growth factors, A-II, and reactive oxygen species, PaSCs promote fibroblastic proliferation, contributing to the desmoplastic stroma that infiltrates and envelops PDAC (100). In mouse models fed a high-fat, high-calorie diet, increased PaSC activation was associated with high stromal fibrosis, a robust inflammatory response, and PanIN progression (101).

Obesity elevates insulin and insulin-like growth factor 1 levels, stimulating PaSC growth and extracellular matrix production, further promoting fibrogenesis, desmoplasia, and PDAC progression (102,103). In addition, WNT5a protein, increased in obesity, promotes proliferation, migration, and invasion of cancer cells and is involved in epithelial-to-mesenchymal transition (104). In cocultures of 3T3-L1 adipocytes and MiaPaCa-2 PDAC cell line, WNT5a induced mature adipocytes to dedifferentiate and reprogram into fibroblast-like cells (105). Thus, obesity-mediated activation of PaSCs might contribute to tumor progression by promoting fibrogenesis, desmoplasia, and enhanced cell migration. Nonetheless, adipocytes have also been shown to promote the *in vitro* proliferation of PanIN and PDAC by supplying glutamine under nutrient-deprived conditions characteristic of the tumor microenvironment, suggesting a potential mechanism for pancreatic steatosis-induced PDAC independent of obesity (106).

### Mitophagy and oxidative stress

Mitophagy, a specialized form of macroautophagy essential for mitochondrial quality control, is disrupted in various diseases (107–109). In pancreatic steatosis, accumulated nonesterified fatty acids and triglycerides cause oxidative stress, mitochondrial uncoupling, inflammation, and cell death (38,110). In murine models, a high-fat, high-sucrose diet promoted pancreatic steatosis, oxidative stress, and β-cell apoptosis (111). The high expression of autophagy-related proteins, such as p62, WDFY3, and NQO1, in acinar cells near pancreatic steatosis, especially in acinar-ductal metaplasia and pancreatic cancer cells, indicates a link between autophagy disruption, oxidative stress, DNA damage, and pancreatic cancer (112). Hyperinsulinemia-induced oxidative stress, along with deregulated iron homeostasis and hyperferritinemia, hallmarks of dysmetabolic iron overload syndrome observed in approximately one third of patients with MASLD, contribute to oxidative stress (38,113–116). Although hyperferritinemia is associated with pancreatic steatosis, malignant IPMNs, and PDAC, its specific role in pancreatic steatosis-induced PDAC requires further investigation (55,117–119).

## CLINICAL IMPLICATIONS

### Role of pancreatic steatosis in PDAC surveillance

The role of pancreatic steatosis in PDAC surveillance remains unclear. The US Preventive Services Task Force does not recommend routine surveillance for asymptomatic individuals at average risk of pancreatic cancer (120). Current guidelines suggest surveillance for high-risk individuals, defined as those with at least a 5% lifetime risk of developing pancreatic cancer, based on family history and germline variant status (121). Surveillance protocols for those high-risk individuals include EUS, MRI and/or magnetic retrograde cholangiopancreatography, and glucose testing to be performed annually, provided that no concerning lesions are detected (121). Surveillance aims to identify early resectable stage I PDAC and high-risk precursor neoplasms (88). Although pancreatic steatosis is a risk factor for PDAC, it remains unclear which patients with pancreatic steatosis might benefit from surveillance. In the absence of specific guidelines, introducing surveillance for higher-risk patients, considering factors like the severity and duration of steatosis and additional high-risk features, may be necessary. The need to define whether pancreatic steatosis detection in high-risk individuals should prompt immediate PDAC investigation, as happens with new-onset diabetes, or shorter follow-up intervals remains critical (88).

### Therapeutics

Pancreatic steatosis is reversible through lifestyle modifications, such as a Mediterranean diet, intermittent fasting, regular exercise, and smoking cessation (122–126). Bariatric surgery also reduces pancreatic steatosis, yet its impact on PDAC risk remains unclear (127–129). A large retrospective study involving 22,198 patients who underwent bariatric surgery found a 54% reduction in the risk of pancreatic cancer after surgery (130). Currently, no specific medications exist for the treatment of pancreatic steatosis and research on the effects of glycemic control agents and lipid-lowering drugs is limited. A small study found that 16 weeks of metformin treatment did not reduce pancreatic steatosis (131). Glucagon-like peptide-1 agonists showed no benefit (132,133). However, liraglutide, but not with glimepiride, reduces steatosis among patients with type 2 diabetes mellitus (134). Atorvastatin reduced pancreatic steatosis and ER stress in obese mice (135). Recent research suggests potential links between pancreatic steatosis, microbiota metabolites, and serum uric acid levels, highlighting potential therapeutic targets (136,137). Furthermore, the role of renin-angiotensin system manipulation in pancreatic steatosis-induced PDAC remains to be explored.

## CONCLUSIONS

Large-scale population studies are crucial to accurately characterize the epidemiology of pancreatic steatosis, which, despite limited research, is a highly prevalent condition. Establishing consensus on optimal diagnostic methods, cutoffs, and severity grading is essential. Inflammatory processes, oxidative stress, and alterations in the local microenvironment may contribute to pancreatic steatosis-induced PDAC development. However, significant gaps remain in understanding how pancreatic steatosis specifically increases PDAC risk. Clarifying these mechanisms is vital for developing effective targeted interventions. Prospective studies are needed to explore the role of pancreatic steatosis in early cancer detection and intervention and to address clinical gaps in understanding its role in PDAC surveillance. Finally, investigating the impact of reversing pancreatic steatosis on PDAC risk is critical for identifying potential preventive strategies for PDAC.

## CONFLICTS OF INTEREST

**Guarantor of the article:** Christos Fountzilas, MD.

**Specific author contributions:** Z.P.: writing—original draft and investigation. P.D.: writing—review and editing (supporting). R.F.: writing—review and editing (supporting) and software. C.F.: supervision, funding acquisition, and writing—review and editing (supporting).

**Financial support:** C.F. receives support from the NIH R37CA282430 grant and the Roswell Park Comprehensive Cancer Center Support Grant P30CA016056.

**Potential competing interests:** None to report.
